# Long-term efficacy and safety of SARS-CoV-2 vaccination in patients with chronic kidney disease, on dialysis or after kidney transplantation: a national prospective observational cohort study

**DOI:** 10.1186/s12882-022-02680-3

**Published:** 2022-02-05

**Authors:** P. Bouwmans, A. L. Messchendorp, J. S. Sanders, L. Hilbrands, M. E. J. Reinders, P. Vart, F. J. Bemelman, A. C. Abrahams, M. A. van den Dorpel, M. A. Ten Dam, A. P. J. de Vries, T. Rispens, M. Steenhuis, R. T. Gansevoort, M. H. Hemmelder, RECOVAC Collaborators, Marcia L. Kho, Debbie van Baarle, Renate G. van der Molen, Carla C. Baan, Dimitri A. Diavatopoulos, Ester B. M. Remmerswaal, Celine Imhof, Reshwan S. R. K. Malahe, Sophie C. Frölke, Nynke Rots, Fiona van der Klis, Ester ten Hoope, Wanda S. Konijn, Tony de Ronde, Johanna P. M. Vervoort, Marion H. J. Braks

**Affiliations:** 1 Department of Internal Medicine, Division of Nephrology, Maastricht University Medical Center, Maastricht, the Netherlands; 2 CARIM school for cardiovascular disease, University of Maastricht, Maastricht, the Netherlands; 3 Department of Nephrology, University Medical Center Groningen, University of Groningen, Groningen, the Netherlands; 4 Department of Nephrology, Radboud University Medical Center, Nijmegen, the Netherlands; 5 Internal Medicine, Nephrology and Transplantation, Erasmus MC Transplant Institute, Erasmus Medical Center, Rotterdam, the Netherlands; 6 Department of Internal Medicine, University Medical Center Groningen, Groningen, the Netherlands; 7 Department of Clinical Pharmacy and Pharmacology, University Medical Center Groningen, Groningen, the Netherlands; 8 Department of Internal Medicine, Division of Nephrology, Amsterdam University Medical Center – location Amsterdam Medical Center, Amsterdam, the Netherlands; 9 Department of Nephrology and Hypertension, University Medical Center Utrecht, Utrecht, the Netherlands; 10 Department of Nephrology, Maasstad Hospital, Rotterdam, the Netherlands; 11 Department of Internal Medicine, Canisius Wilhelmina Hospital, Nijmegen, the Netherlands; 12 Department of Medicine, Division of Nephrology, and Leiden Transplant Center, Leiden University Medical Center, Leiden, the Netherlands; 13 Department of Immunopathology, Sanquin Research, Amsterdam, The Netherlands; 14 Landsteiner Laboratory, Amsterdam University Medical Center, University of Amsterdam, Amsterdam, The Netherlands

**Keywords:** SARS-CoV-2, Vaccine, Chronic kidney disease, Kidney transplantation, Dialysis, Safety, Efficacy, Antibody response

## Abstract

**Background:**

COVID-19 is associated with increased morbidity and mortality in patients with chronic kidney disease (CKD) stages G4-G5, on dialysis or after kidney transplantation (kidney replacement therapy, KRT). SARS-CoV-2 vaccine trials do not elucidate if SARS-CoV-2 vaccination is effective in these patients. Vaccination against other viruses is known to be less effective in kidney patients. Our objective is to assess the efficacy and safety of various types of SARS-CoV-2 vaccinations in patients with CKD stages G4-G5 or on KRT.

**Methods:**

In this national prospective observational cohort study we will follow patients with CKD stages G4-G5 or on KRT (*n* = 12,000) after SARS-CoV-2 vaccination according to the Dutch vaccination program. Blood will be drawn for antibody response measurements at day 28 and month 6 after completion of vaccination. Patient characteristics and outcomes will be extracted from registration data and questionnaires during 2 years of follow-up. Results will be compared with a control group of non-vaccinated patients. The level of antibody response to vaccination will be assessed in subgroups to predict protection against COVID-19 breakthrough infection.

**Results:**

The primary endpoint is efficacy of SARS-CoV-2 vaccination determined as the incidence of COVID-19 after vaccination. Secondary endpoints are the antibody based immune response at 28 days after vaccination, the durability of this response at 6 months after vaccination, mortality and (serious) adverse events.

**Conclusion:**

This study will fulfil the lack of knowledge on efficacy and safety of SARS-CoV-2 vaccination in patients with CKD stages G4-G5 or on KRT.

**Trial registration:**

The study protocol has been registered in clinicaltrials.gov(NCT04841785).

**Current knowledge about this subject**

- COVID-19 has devastating impact on patients with CKD stages G4-G5, on dialysis or after kidney transplantation.
- Effective SARS-CoV-2 vaccination is very important in these vulnerable patient groups.
- Recent studies on vaccination in these patient groups are small short-term studies with surrogate endpoints.

**Contribution of this study**

- Assessment of incidence and course of COVID-19 after various types of SARS-CoV-2 vaccination during a two-year follow-up period in not only patients on dialysis or kidney transplant recipients, but also in patients with CKD stages G4-G5.
- Quantitative analysis of antibody response after SARS-CoV-2 vaccination and its relationship with incidence and course of COVID-19 in patients with CKD stages G4-G5, on dialysis or after kidney transplantation compared with a control group.
- Monitoring of (serious) adverse events and development of anti-HLA antibodies.

**Impact on practice or policy**

- Publication of the study design contributes to harmonization of SARS-CoV-2 vaccine study

methodology in kidney patients at high-risk for severe COVID-19.

- Data on efficacy of SARS-CoV-2 vaccination in patients with CKD will provide guidance for future vaccination policy.

**Supplementary Information:**

The online version contains supplementary material available at 10.1186/s12882-022-02680-3.

## Background

The coronavirus disease 2019 (COVID-19) pandemic has great impact on all aspects of society and healthcare. Patients with a severely impaired kidney function (CKD stages G4-G5) and patients on dialysis or after kidney transplantation, referred to as kidney replacement therapy (KRT), were shown to be extremely vulnerable. The COVID-19 associated mortality risk in these patient groups was reported to be 3- to 4-fold increased as compared to the general healthy population [1], which is considerably higher than the 1.5-to-2-fold increase that was described in patients with obesity, hypertension or diabetes [2]. Data from the European Renal Association – European Dialysis and Transplant Association (ERA-EDTA) COVID-19 database (ERACODA) show a high case fatality rate in patients on KRT [3, 4]. Therefore, the availability of an effective and safe vaccine will be extremely important for patients with CKD stages G4-G5 or on KRT.

Recently, efficacy and safety of different SARS-CoV-2 vaccine types have been demonstrated to be high in the general population [5–8]. So far, patients with CKD stages G4-G5 or on KRT were not or in low numbers included in these trials [9]. It is well known that vaccination efficacy against other viruses such as hepatitis B and influenza is considerably lower in patients with severely impaired kidney function, due to the immunosuppressive effect of uremic waste products [10]. A lesser antibody response after vaccination is to be expected in kidney transplant recipients due to the use of immunosuppressive agents. A recent Call to Action by the ERA-EDTA Council and ERACODA Working Group included the advice to perform dedicated vaccination studies to address this important knowledge gap regarding the efficacy of SARS-CoV-2 vaccination [11]. Recent studies have demonstrated the humoral response following SARS-CoV-2 vaccination in patients on KRT. One study showed a robust humoral response in the majority of haemodialysis patients after SARS-CoV-2 vaccination [12], but in a different study when compared to a control group, antibody titres were significantly lower [13]. Another study demonstrated a significantly reduced antibody response in 34 solid organ transplantation patients compared to health care workers [14]. Current data on SARS-CoV-2 vaccination efficacy in kidney patients report on surrogate endpoints, such as antibody titres. Clinical endpoints are currently lacking in the assessment of the efficacy of SARS-CoV-2 vaccination in kidney patients.

Therefore, we established the RECOVAC consortium (**RE**nal patients **CO**vid-19 **VAC**cination) in the Netherlands with active participation of the Dutch Kidney Patients Association (NVN). This consortium will perform the **L**ong-term **E**fficacy and **S**afety of **S**ARS-**CoV-2** vaccination (LESS CoV-2) study. In this study we aim to assess efficacy of SARS-CoV-2 vaccination in patients with CKD stages G4-G5 and on KRT in comparison to non-vaccinated control groups in a large-scale national registry study. We hypothesize that kidney patients obtain lesser protection from SARS-CoV-2 vaccination.

## Methods and analysis

### Study design

This is a prospective national observational cohort study which includes all kidney patients who were vaccinated against SARS-CoV-2 in the Dutch vaccination programme. Currently available vaccines in the Netherlands are the mRNA-1273 (Moderna), BNT162b2-mRNA (Pfizer), ChAdOx1 nCoV-19 (AstraZeneca) and Ad26.COV2.S (Janssen) vaccines [5–8]. Patients with CKD stages G4-G5 or on KRT received prioritization as medical high-risk group and predominantly received the mRNA-1273 vaccine in the Dutch vaccination programme. To assess the antibody dependent immune response after vaccination, blood samples will be collected at 28 days and at 6 months after the second vaccination with a home-based finger prick test. Antibody measurement will include both the receptor binding domain (RBD) of the spike (S) antigen and the nucleocapsid (N) antigen [15]. Combining these antibody measurements allows to distinguish a natural infection from an antibody response after S1-based vaccination.

**Fig. 1:**
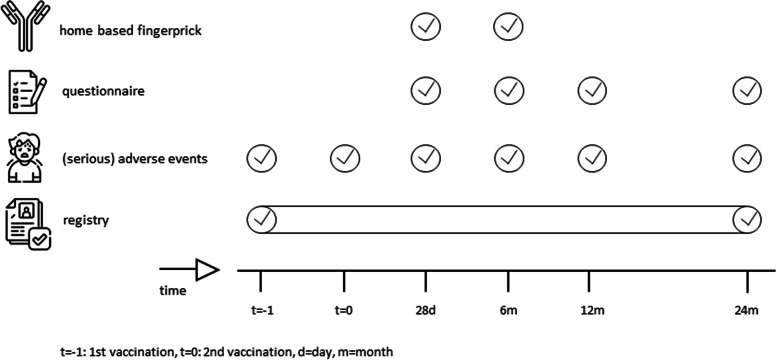
Study activities. “t = -1” = 1st vaccination, “t = 0” = 2nd vaccination, d = days, m = months*.* Details on the course of COVID-19 after vaccination will be collected from ERACODA which collects individual patient level data of adults on dialysis or with a functioning kidney allograft who are diagnosed with COVID-19

Questionnaires will be obtained during a two-year follow-up period to assess the incidence of COVID-19 or (serious) adverse events ((S)AEs) after vaccination. The study events are demonstrated in Fig. 1. The questionnaires are shown in the supplemental materials.

### Setting

The study starts in April 2021 amidst the COVID-19 pandemic when the Dutch vaccination program has started to vaccinate high-risk patients, including patients with kidney disease. COVID-19 incidence rates are still high and the health care system has not yet regained full capacity to exert routine care to all patients, due to lack of capacity.

### Study participants

The study population includes a subset of patients with CKD stages G4-G5 and patients on KRT in the Netherlands. The inclusion and exclusion criteria are mentioned in Table 1. Of note, patients will be eligible whether they have a history of COVID-19 or not.

**Table 1:** Inclusion and exclusion criteria

*Inclusion criteria*	
1. Age of 18-80 years 2. Capable of understanding the purpose and risks of the study, to be fully informed and to give informed consent 3. Either - eGFR < 30 ml/min/1.73m^2^ but not on dialysis or with a kidney transplant - Haemodialysis or peritoneal dialysis - Kidney transplant recipient at least 6 weeks after transplantation	
*Exclusion criteria (≥ 1)*	
•Patients who opted out for the national dialysis (RENINE) and kidney transplantation (NOTR) registries.	

*eGFR* Estimated glomerular filtration rate

### Study enrolment

All patients who previously gave informed consent in two national registries, for dialysis patients (RENINE) and kidney transplant recipients (NOTR), are selected for enrolment. Patients with CKD stages G4-G5 will be enrolled from a cohort of non-university hospitals (Santeon). All patients eligible to the registry will be followed to assess incidence of COVID-19 after vaccination, (S)AEs and vaccination status. Data of non-vaccinated patients which will be derived from RENINE and NOTR registries serve as control. Furthermore, data on vaccination efficacy in the general population, which will be derived from the National Institute for Public Health and the Environment (RIVM), serve as a control group.

**Fig. 2:**
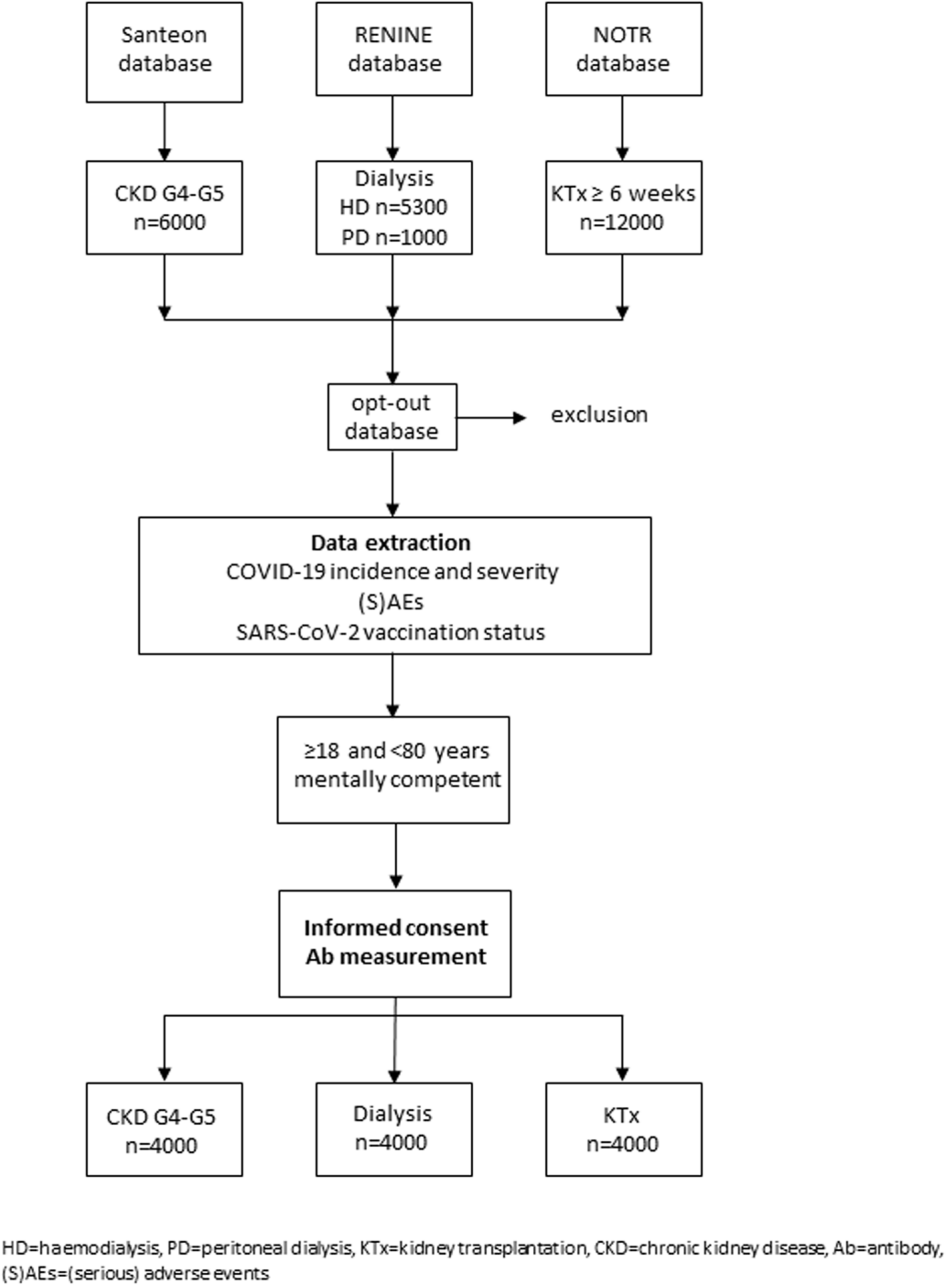
Study Enrolment Flowchart. HD = haemodialysis, PD = peritoneal dialysis, KTx = kidney transplantation, CKD = chronic kidney disease, Ab = antibody, (S)AEs = (serious) adverse events

For measurement of antibodies after completion of SARS-CoV-2 vaccination we intend to include a random subset of patients with CKD stages G4-G5 (*n* = 4000), patients on dialysis (*n* = 4000), and patients after kidney transplantation (*n* = 4000) (Fig. 2). Patients older than 80 years are excluded from participation because they had priority in the Dutch national vaccination scheme over other high-risk groups. Consequently, they were vaccinated longer than 28 days ago, which is the accepted term in wherein finger-prick tests are obtained for antibody measurement.

### Informed consent for antibody measurement at home

All patients eligible to antibody measurement at home are offered two options to give informed consent, namely a written and a digital informed consent. We invited patients to register for the study by e-mail, mail and by distribution of flyers in all participating hospitals. Patients could apply for a patient information folder being sent to their home address or they could access the information folder at the website by scanning a QR-code. The QR-code directed the patients to a digital platform where they could give a digital informed consent, signed by entering a code sent to their mobile phone. In addition, we used our study website [16] to provide online access to the digital platform where informed consent could be given. This procedure enables rapid information provision of patients with minimal effort for local health care workers in the hospitals.

### Patient characteristics

The patient characteristics that will be obtained are listed in Table 2.

**Table 2:** Patient baseline characteristics

*General*	
• Age • Sex • BMI	
*Renal disease*	
• Primary kidney disease • Dialysis modality • Type of kidney transplantation.	
*Laboratory parameters*	
• Serum creatinine (μmol/L), eGFR (ml/min1.73m^2^), except in dialysis patients • Urine creatinine (g/L), urine protein (g/L) or albumin (g/L), except in dialysis patients • HbA1c (mmol/mol)	
*Intoxications*	
• Smoking, alcohol	
*Comorbidity*	
• Coronary heart disease / PCI / CABG • Heart failure • Hypertension • Peripheral arterial disease and/or abdominal aortic aneurysm • Cerebrovascular accident • Dementia • Lung disease • Liver disease • Diabetes • Cancer • HIV	
*PROMs*	
• SF-12 and DSI in the subset of dialysis patients	
*Medication*	
• Antihypertensive drugs • Steroids or other immunosuppressive agents	
*COVID-19*	
• COVID-19 and severity before vaccination	

*BMI* Body Mass Index, *eGFR* estimated Glomerular Filtration Rate, *HbA1c* Haemoglobin A1c, *PCI* Percutaneous Coronary Intervention, *CABG* Coronary Arterial Bypass Grafting, *HIV* Human Immunodeficiency Virus, *PROMs* Patient Reported Outcome Measures, *SF-12* 12-item Short Form health survey, *DSI* Dialysis symptom index, *COVID-19* Coronavirus disease 2019

### Outcome definitions

For an overview of the clinical endpoints in this study we refer to Table 3.

**Table 3:** Clinical endpoints LESS-CoV-2 study

*Primary endpoint*	
Incidence (per person-year) of COVID-19 in a two-years period after SARS-CoV-2 vaccination in patients with CKD stage G4-G5 or on KRT.	
*Secondary endpoints*	
Efficacy	
• The antibody based immune response to SARS-CoV-2 vaccination at 28 days after SARS-CoV-2 vaccination.	
• The durability of the antibody response at 6 months after SARS-CoV-2 vaccination	
Safety	
• Incidence of mortality	
• Incidence of AEs of specific interest as defined by (inter) national authorities	
• Incidence of a combined endpoint of acute rejection or graft failure in kidney transplant recipients	
• Incidence of HLA antibodies defined as cPRA > 5% in dialysis patients on the waiting list for their first kidney transplantation	
*Exploratory endpoints*	
Vaccination coverage rates	
Disease severity in patients who develop COVID-19, assessed as	
• Hospitalization	
• ICU admission	
• Mechanical ventilation	
PROMs in patients on dialysis	
SARS-CoV2 genotype in patients with COVID-19 (in case available)	

*COVID-19* Coronavirus Disease 2019, *SARS-CoV-2* Severe Acute Respiratory Syndrome Coronavirus-2, *CKD* Chronic Kidney Disease, *KRT* Kidney Replacement Therapy, *AEs* Adverse Events, *HLA* Human Leukocyte Antigen, *cPRA* Calculated Panel Reactive Antibody, *ICU* Intensive Care Unit, *PROMs* Patient Reported Outcome Measures

### Home based finger prick tests

Blood samples will be obtained through self-obtained sample collection by use of a home-based finger prick test. The finger prick tests will be sent by mail to all participants. Written instructions are given to apply the finger prick test and how to return the finger prick tests. This procedure enables blood sampling without further need of effort from local health care workers in the hospitals. Only hemodialysis patients were offered to contact their dialysis center, when they encounter difficulties during the blood sample collection.

### Antibody measurement

We will analyse the presence of antibodies against the RBD of the SARS-CoV-2 S-protein (IgG anti-RBD antibody) using the Sanquin anti-SARS-CoV-2 RBD IgG ELISA assay [15]. This is an indirect ELISA using microtiter plates coated with RBD and detection by monoclonal mouse anti-human IgG. The antibodies against the RBD are the primary constituent of the humoral immune response after SARS-CoV-2 vaccination. We combine this test with a Sanquin anti-SARS-CoV-2 nucleocapsid protein (NP) bridging ELISA. This test is an indirect ELISA using microtiter plates coated with NP and detection by biotin-labelled NP. Both antibodies against RBD and NP arise after a natural COVID-19 infection. In contrast, vaccination only induces antibodies to RBD, since NP is not part of any of the currently used vaccines. Combining these two tests enables us to differentiate between antibodies after vaccination or after previous COVID-19 infection.

### SARS-CoV-2 genotype variants

We intend to collect data on SARS-CoV-2 genotype variance in our study population if available from clinical practice. We aim to assess the efficacy of the various SARS-CoV-2 vaccine types used in our study population for protection against different SARS-CoV-2 strains.

### Statistical considerations

Baseline characteristics of vaccinated patients with CKD stages G4-G5 or on KRT will be compared with non-vaccinated patients with CKD stages G4-G5 or on KRT and the vaccinated general population using a two-sample t-test in case of continuous variables (or a Mann-Whitney U-test if data are not normally distributed), and a Pearson Chi-2 test in case of categorical variables.

#### Analysis of primary endpoint

Incidence rates of COVID-19 within 2 years after vaccination will be reported for each study population separately and incidence rates will be compared. Primary comparisons are comparisons of COVID-19 incidence rates in vaccinated patients versus non-vaccinated patients and versus the vaccinated general population. These comparisons will be made separately for patients with CKD stages G4-G5 or on KRT. For comparison of incidence rates, we will calculate crude hazard ratios. Vaccine efficacy will be reported as 100 × (1- hazard ratio). We will calculate model adjusted hazard ratios using multivariable Cox-regression, in case of imbalance in study populations regarding relevant characteristics, such as age, sex, comorbidity, kidney replacement modality and use of immunosuppressive agents. Cause specific hazard ratios will be estimated to account for competing risk of mortality. Starting date of follow-up will be the date of second vaccination in vaccinated patients. Starting date of follow-up in non-vaccinated patients will be the same date (or +/− 7 days from date of vaccination) as date of vaccination in the matched vaccinated patients to account for changes in incidence of COVID-19 over time. Non-vaccinated patients will be censored at the day of vaccination in case they are vaccinated before the end of follow-up. The aforementioned analyses will be repeated in patients with and patients without evidence of prior infection. Among vaccinated patients, incidence rates will also be compared across the various vaccines that will be used. For this purpose, vaccinated patients with CKD stages G4-G5 or on KRT and general population again will be analysed separately.

#### Analysis of secondary endpoints

##### Efficacy - Antibody response

Participants will be classified as responders or non-responders [17]. Number and proportion of responders and non-responders at day 28 will be reported for each patient population. Pearson chi-2 test will be performed to assess whether antibody response differ between patient groups. In subsequent sensitivity analyses we will perform multiple regression analyses correcting for possible differences in age and sex between the patient groups. Additionally, in kidney transplant recipients we will investigate whether time after transplantation, and the different immunosuppressive regimens are associated with response rate.

To assess durability of response within each patient group, we will report and compare response at day 28 with response at month 6 after vaccination. Comparison will be made using McNemar test.

The association between antibody response after SARS-CoV-2 vaccination and protection against COVID-19 will be examined for each patient group separately. For this, we will compare the antibody response at 28 days after vaccination in patients who develop COVID-19 with the antibody response in patients who did not develop COVID-19. Similar comparison will be made at 6 months after vaccination. We will examine this by use of the t-test or if more appropriate a non-parametric test. Subsequently, in a logistic regression model using Youden’s index, a cut-off in the antibody response at 28 days will be defined to serve as a correlate of protection against COVID-19.

##### Safety

Incidence rates of (S)AEs, mortality, acute rejection, graft failure or HLA antibodies will be calculated and compared across patient groups in analyses similar to analysis of the primary outcome.

## Discussion

In the LESS-CoV-2 study we will assess the efficacy and safety of the mRNA-1273, BNT162b2, ChAdOx1 nCoV-19 and Ad26.COV2.S SARS-CoV-2 vaccines in Dutch patients with CKD stages G4-G5, on dialysis or after kidney transplantation.

We expect to determine an antibody response cut-off value which allows to predict protection against COVID-19 breakthrough infection after completion of SARS-CoV-2 vaccination. This contributes to the establishment of surrogate endpoints which will facilitate the development and approval of new vaccines [18]. In this study, we will identify non-responders by means of an insufficient antibody response after SARS-CoV-2 vaccination. This population can be offered a booster vaccine or an alternative vaccine to assess whether or not a sufficient antibody response can still be achieved.

Furthermore, we will address the safety of SARS-CoV-2-vaccination in patients with severe kidney insufficiency and in those that are treated with immunosuppressive drugs. Since these patients were not sufficiently represented in the large registration studies of the various vaccines, specific AEs associated with kidney failure and its treatment may not have been recognized so far. In addition, rare but possibly harmful AEs must be searched for in pharmacovigilance studies after widespread distribution of the vaccines. Recent reporting of vaccine-induced immune thrombotic thrombocytopenia caused by the ChAdOx1 nCoV-19 and Ad26.COV2.S SARS-CoV-2 vaccines is a possible example of such a rare complication [19]. It is essential to collaborate with large registry studies to be able to detect rare SAEs in patients with kidney disease. Different SARS-CoV-2 vaccines are administered to our subjects, and for that reason we will monitor the development of immunisation against HLA-antigens, allograft rejection and/or kidney transplant failure following SARS-CoV-2 vaccination.

Considering that items such as mental QoL, anxiety and depression may be positively influenced by vaccination, it is highly relevant to monitor the effect of SARS-CoV-2 vaccination on patient reported outcome measurements (PROMs). In our cohort of dialysis patients PROMs have been monitored since 2019. During the study we will further monitor the course of mental health of vaccinated and non-vaccinated dialysis patients and compare this to pre-COVID-19 pandemic era and the COVID-19 pandemic era without vaccines.

In our study we encountered the challenge to approach a large population to provide informed consent and obtain blood samples under high time pressure. We provided a variety of tools to overcome possible thresholds. First, the digital informed consent gave patients the opportunity to readily access study information and participation. An alternative option to apply for written informed consent was provided, thereby including patients that are not able to access digital platforms. Second by using a home-based finger prick test for antibody measurement, patients were not obliged to make travel expense to obtain blood samples and local health care workers were not burdened with more work during the pandemic. In addition, high-risk patient groups will not be exposed to an additional risk of contamination with SARS-CoV-2 due to extra visits to the hospitals. This strategy may attribute to a higher participation grade.

While our study has a real-life observational design, there are some relevant issues to mention which may impact the results. The reported efficacy depends on the presence of widespread contamination. If for any reason widespread contamination is halted, efficacy of vaccination might be confounded, hence less contamination will occur. The opposite could be the case in the midst of a pandemic where widespread contamination occurs. Additionally, participants are vaccinated according to the national Dutch vaccination programme, in which mRNA-1273 is predominantly administered to high-risk groups, including patients on dialysis or after kidney transplantation. Consequently, certain types of vaccines might be underrepresented. In this study we will only assess the humoral response following SARS-CoV-2 vaccination. Therefore, the immunogenicity of SARS-CoV-2 vaccination in high-risk kidney patients cannot be fully assessed.

Nonetheless, the LESS CoV-2 study will provide key information with regards to the efficacy of SARS-CoV-2 vaccines in high-risk kidney patients. Antibody response data and clinical endpoints will be collected in a large cohort of patients of whom patient characteristics uniformly are obtained in national registries with almost complete coverage. All high-risk kidney patient groups are represented in the study. As such, the study will be sufficiently powered to identify patients’ characteristics and the humoral antibody response predisposing for COVID-19 breakthrough infection. Our study can serve as a template for other studies of vaccine efficacy in high-risk populations including the admission of digital informed consent and finger prick blood sampling at home. Harmonizing the design of vaccination studies will allow international comparison of larger datasets.

## Supplementary Information

**Additional file 1.** Questionnaire at 1 month after vaccination.

**Additional file 2.** Questionnaires at 6, 12 and 24 months after vaccination.

## Data Availability

Not applicable.

## Abbreviations

Ab: Antibody
AEs: Adverse Events
BMI: Body Mass Index
CABG: Coronary Arterial Bypass Grafting
CKD: Chronic Kidney Disease
COVID-19: Coronavirus Disease 2019
cPRA: Calculated Panel Reactive Antibody
DSI: Dialysis Symptom Index
eGFR: Estimated Glomerular Filtration Rate
ELISA: Enzyme-Linked ImmunoSorbent Assay
ERACODA: European Renal Association COVID-19 Database
ERA-EDTA: European Renal Association – European Dialysis and Transplant Association
HbA1c: Haemoglobin A1c
HD: Haemodialysis
HIV: Human Immunodeficiency Virus
HLA: Human Leukocyte Antigens
ICU: Intensive Care Unit
IgG: Immunoglobulin G
KRT: Kidney Replacement Therapy
KTx: Kidney Transplantation
LAREB: Netherlands Pharmacovigilance Center
LESS CoV-2: Long term Efficacy and Safety of SARS-CoV-2 vaccination
mRNA: Messenger-RNA
N: Nucleocapsid
NFN: Dutch Federation of Nephrology
NOTR: Dutch kidney transplant recipient registration
NP: Nucleocapsid Protein
NTV: Dutch Transplant Society
NVN: Dutch Kidney Patient Association
PCI: Percutaneous Coronary Intervention
PD: Peritoneal Dialysis
PROMs: Patient-Reported Outcome Measures
QoL: Quality of Life
QR-code: Quick Response code
RBD: Receptor Binding Domain
RECOVAC: REnal patients COvid-19 VACcination
RENINE: Dutch dialysis patient registration
RIVM: Dutch National Institute for Public Health and the Environment
SAEs: Serious Adverse Events
SARS-CoV-2: Severe Acute Respiratory Syndrome Coronavirus-2
S: Spike
SD: Standard Deviation
SF-12: 12-item Short Form Health Survey 12
S1: Spike protein subunit 1
WMA: World Medical Association
WMO: World Medicine Organisation
ZonMw: The Netherlands Organisation for Health Research and Development
