# Supplementary material for: Long-term efficacy and safety of SARS-CoV-2 vaccination in patients with chronic kidney disease, on dialysis or after kidney transplantation: a national prospective observational cohort study
Source: BMC Nephrol. 2022 Feb 5;23:55. doi: 10.1186/s12882-022-02680-3 (PMC8817171; doi:10.1186/s12882-022-02680-3)
Supplement: Supplementary file 1 — Additional file 1. Questionnaire at 1 month after vaccination. [file 12882_2022_2680_MOESM1_ESM.pdf]

Questionnaire LESS CoV-2 study

Long term efficacy and safety of SARS-CoV-2 vaccination in kidney patients

Participation number	
Date	

Would you please fill in and return this questionnaire in the return envelope within 7 days after receiving the questionnaire? (stamp is not required).

Questions?

You can find answers to frequently asked questions on the page of www.recovac.nl. If you cannot find your answer, please send an e-mail to info@recovac.nl or call +3150-3610881 (available Monday to Thursday from 9AM to 12AM).

Instructions for answering the questions

With this questionnaire we would like to know if you have been through COVID-19, which corona vaccine has been given to you and if you have experienced any side effects after vaccination. Besides, we would like to ask you questions regarding your health. For example, we would like to know whether you use any kind of medication and if you have been diagnosed with diseases other than kidney disease. You can answer most questions by choosing from several options. Please fill in the option that applies to your situation.

- Answer all questions. You can skip a question only if it is specifically stated that the question does not apply to you.
- Per question you can fill in one option. You can choose more options only when it is specifically stated that you are allowed to do so.
- If an answer option is followed by dots, you are expected to fill in your answer there.
- Please use a pen with black or dark blue ink.
- To be clear, this is not a test; there are no ‘correct or ‘wrong’ answers.

The following questions are about general aspects of your body, health and medicine use

1. What is your body length? ………………………………

2. Wat is your body weight (estimated) (kg)? ………………………………

3. Do you smoke?
   - ☐ Yes
   - ☐ In the past
   - ☐ Never

4. Do you consume alcohol?
   - ☐ No, (nearly) never
   - ☐ Less than daily
   - ☐ Daily

5. Do you use any of the following medicine?
   Multiple answers can apply
   - ☐ Antihypertensive medicine
   - ☐ Blood sugar lowering medicine
   - ☐ Prednisolone
   - ☐ Mycophenalate Mofetil (also referred to as CellCept, Myfortic, Myfenax)
   - ☐ Azathioprin (also reffered to as Imuran)
   - ☐ One of the following medicines: tacrolimus (also referred to as Adport, Advagraf, Dailiport, Envarsus, Modigraf, Prograft) or ciclosporin (also referred to as Neoral)
   - ☐ One of the following medicines: everolimus (Certican) or sirolimus (Rapamune)
   - ☐ No, none of the above

6. Are you known with one of the following diseases or have you had any of these previously?
   Multiple answers are possible
   - ☐ Coronary infarction or previous stent placement or bypass surgery of the heart
   - ☐ Blood vessel disease of the abdomen artery (aorta) and/or legs
   - ☐ Heart failure
   - ☐ Diabetes
   - ☐ Hypertension
   - ☐ Cancer, treated with chemo- or immunotherapy in the past five years
   - ☐ Stroke
   - ☐ Dementia
   - ☐ Lung diseases, such as emphysema, asthma or COPD
   - ☐ Liver cirrhosis
   - ☐ HIV/aids
   - ☐ No, none of the above.

7. What is your origin?
   - ☐ European
   - ☐ Sahara (Maroc, Algeria, Tunisia, Libya, Egypt)
   - ☐ Sub-Saharan Africa (other African countries)
   - ☐ Middle East (including Turkey)
   - ☐ Asian
   - ☐ Creole or Afro-Caribbean
   - ☐ Latin-American
   - ☐ Multiple descent
   - ☐ I do not know
   - ☐ I rather not share
   - ☐ Other, ….

<u>The following questions are about the period before your vaccination</u>

8. Have you been tested positive for the coronavirus?
   - ☐ Yes
   - ☐ No *Please proceed to question 13*

9. When have you been tested positive for the coronavirus? (mm/yyyy)?

   ____ / ____

10. Have you experienced any symptoms of an infection with the coronavirus?
    - ☐ Yes
    - ☐ No

11. Have you been admitted to the hospital due to a coronavirus infection?
    - ☐ Yes
    - ☐ No *Please proceed to question 13*

12. Have you been admitted to an ICU due to a coronavirus infection?
    - ☐ Yes
    - ☐ No

<u>The following questions are about the corona vaccination you have been given.</u>

13. Which type of corona vaccine has been given to you?
    - ☐ Moderna
    - ☐ Pfizer
    - ☐ AstraZeneca
    - ☐ Janssen
    - ☐ Other, …..
    - ☐ Do not know

14. When have you been vaccinated?
If you cannot recall the exact date, please fill in only the month and the year of vaccination.

- ☐ Date 1st vaccination (dd/mm/yyyy)
  ____ /____ / ____
- ☐ If applicable, date 2nd vaccination (dd/mm/yyyy)
  ____ /____ /____

The following questions are about the period after vaccination

15. Have you experienced any side effects within 7 days after the 1st vaccination?
Multiple answers can apply
- ☐ Allergic reaction within 15 minutes after vaccination
- ☐ Fever (body temperature > 38 degrees Celsius)
- ☐ Muscle strain
- ☐ Joint complaints
- ☐ Pain in the arm at the site of injection
- ☐ Thrombosis
- ☐ No
- ☐ Other, ….
- ☐ Unknown

16. Have you experienced any side effects within 7 days after the 2nd vaccination.
Multiple answers can apply
- ☐ Allergic reaction within 15 minutes after vaccination
- ☐ Fever (body temperature > 38 degrees Celsius)
- ☐ Muscle strain
- ☐ Joint complaints
- ☐ Pain in the arm at the site of injection
- ☐ Thrombosis
- ☐ No
- ☐ Other, ….
- ☐ Unknown

17. Have you been tested positive for corona after receiving your 1st or 2nd vaccination?
- ☐ Yes, I have been tested positive for an infection with the coronavirus
- ☐ No, I have not been tested positive for an infection with the coronavirus
  Please proceed to question 24.

18. When have you been tested positive for corona? (mm/yyyy) ____ / ____

19. Where were you tested?
    Multiple answers can apply
    - ☐ Municipal Health Services
    - ☐ Hospital
    - ☐ Commercial provider
    - ☐ General practitioner
    - ☐ At home – self-test
    - ☐ Other, …..
    - ☐ Do not know

20. With what kind of test have you been tested?
    - ☐ An extensive test (PCR-test), of which I have received a result within 1-2 days.
    - ☐ A self-test (antigen test), of which I have received a result within minutes to hours.
    - ☐ Do not know.

21. At the time of coronavirus infection, did you have any symptoms?
    - ☐ Yes
    - ☐ No

22. Have you been admitted to the hospital because of a coronavirus infection.
    - ☐ Yes
    - ☐ No
    - ☐ I have been admitted because of another reason and have been tested positive for the coronavirus during admission.

23. Have you been admitted to the ICU because of a coronavirus infection?
    - ☐ Yes
    - ☐ No
    - ☐ I have been admitted because of another reason and have been tested positive for the coronavirus during admission.

24. Have you been admitted to the hospital because of a reason other than a coronavirus infection?
    - ☐ Yes
    - ☐ No *Please proceed to question 37*

25. If yes, have you been admitted to the hospital more than once?
    - ☐ Yes
    - ☐ No

26. If yes, when have you been admitted? (admission 1) (mm/yyyy) ____ / ____

27. For what reason have you been admitted? (admission 1)
Multiple answers can apply
- ☐ An infection (other than the coronavirus)
- ☐ Heart and vascular disease (for example heart attack or stroke)
- ☐ Surgery / planned operation
- ☐ Other, …..

28. If yes, when have you been admitted? (admission 2) (mm/yyyy) ____ / ____

29. For what reason have you been admitted? (admission 2)
Multiple answers can apply
- ☐ An infection or inflammation (other than the coronavirus)
- ☐ Heart and vascular disease (for example heart attack or stroke)
- ☐ Surgery / planned operation
- ☐ Other, …..

30. If yes, when have you been admitted? (admission 3) (mm/yyyy) ____ / ____

31. For what reason have you been admitted? (admission 3)
Multiple answers can apply
- ☐ An infection (other than the coronavirus)
- ☐ Heart and vascular disease (for example heart attack or stroke)
- ☐ Surgery / planned operation
- ☐ Other, …..

32. If yes, when have you been admitted? (admission 4) (mm/yyyy) ____ / ____

33. For what reason have you been admitted? (admission 4)
Multiple answers can apply
- ☐ An infection (other than the coronavirus)
- ☐ Heart and vascular disease (for example heart attack or stroke)
- ☐ Surgery / planned operation
- ☐ Other, …..

34. If yes, when have you been admitted? (admission 5) (mm/yyyy) ____ / ____

35. For what reason have you been admitted? (admission 5)
Multiple answers can apply
- ☐ An infection (other than the coronavirus)
- ☐ Heart and vascular disease (for example heart attack or stroke)
- ☐ Surgery / planned operation
- ☐ Other, …..

36. After you have been vaccinated, have you been diagnosed with one of the following diseases?
- ☐ Coronary infarction or previous stent placement or bypass surgery of the heart
- ☐ Blood vessel disease of the abdomen artery (aorta) and/or legs

- ☐ Heart failure
- ☐ Diabetes
- ☐ Hypertension
- ☐ Cancer, treated with chemo- or immunotherapy in the past five years
- ☐ Stroke
- ☐ Dementia
- ☐ Lung diseases, such as emphysema, asthma or COPD
- ☐ Liver cirrhosis
- ☐ HIV/aids
- ☐ No, none of the above.

The following questions are about your kidney disease

37. Before you have been vaccinated, which of the following applied to you?
    - ☐ I have a kidney transplantation and I do not dialyse anymore.
      Continue to question 38
    - ☐ I have had a kidney transplantation and I am currently a dialysis patient.
      Continue to question 43
    - ☐ I am currently a dialysis patient and I have never had a kidney transplantation.
      Continue to question 43
    - ☐ I have a kidney function less than 30% and I have never had a kidney transplantation.
      Continue to question 46.

The following questions apply if you had a kidney transplantation before being vaccinated, and if you were not being treated with dialysis.

38. After you have been vaccinated, have you been transplanted again?
    - ☐ Yes
    - ☐ No *Please proceed to question 40*

39. If yes, when? (dd/mm/yyy) ___ / ___ / ___
    If you cannot recall the exact date, please fill in only the month and the year of kidney transplantation.

40. After you have been vaccinated, have you started dialysis treatment?
    - ☐ Yes, haemodialysis
    - ☐ Yes, peritoneal dialysis
    - ☐ No *Please proceed to question 42*

41. If yes, when did you start dialysis treatment? (dd/mm/yyyy) ___ / ___ / ___
    If you cannot recall the exact date, please fill in only the month and the year in which you started dialysis treatment.

42. After you have been vaccinated, did rejection of your kidney transplant occur?
    - ☐ Yes
    - ☐ No

Please proceed to question 50

<u>The following questions apply to you if you were a dialysis patient, before being vaccinated</u>

43. After you have been vaccinated, did anything change in your dialysis modality?
    - ☐ Yes, I have been treated with haemodialysis and recently I have switched to peritoneal dialysis.
    - ☐ Yes, I have been treated with peritoneal dialysis and recently I have switched to haemodialysis.
    - ☐ No

44. After you have been vaccinated, did you receive a kidney transplantation?
    - ☐ Yes
    - ☐ No *Please proceed to question 50*

45. If yes, when? (dd/mm/yyyy) ___ / ___ / ___
    If you cannot recall the exact date, please fill in only the month and the year of kidney transplantation.

    Please proceed to question 50

<u>The following questions apply to you if, before the vaccination, you were not being treated with dialysis and if you did not have a kidney transplant.</u>

46. After you have been vaccinated, have you received a kidney transplantation?
    - ☐ Yes
    - ☐ No *Please proceed to question 48*

47. If yes, when? (dd/mm/yyyy) ___ / ___ / ___
    If you cannot recall the exact date, please fill in only the month and the year of kidney transplantation.

48. After you have been vaccinated, did you start dialysis?
    - ☐ Yes, I have started treatment with haemodialysis
    - ☐ Yes, I have started treatment with peritoneal dialysis
    - ☐ No *Please proceed to question 50*

49. If yes, when? (dd/mm/yyyy) ___ / ___ / ___
    If you cannot recall the exact date, please fill in only the month and the year in which you started dialysis treatment.

<u>End of the questionnaire</u>

50. Date of filling in this questionnaire (dd/mm/yyyy) ___ / ___ / ___
