# Supplementary material for: Long-term efficacy and safety of SARS-CoV-2 vaccination in patients with chronic kidney disease, on dialysis or after kidney transplantation: a national prospective observational cohort study
Source: BMC Nephrol. 2022 Feb 5;23:55. doi: 10.1186/s12882-022-02680-3 (PMC8817171; doi:10.1186/s12882-022-02680-3)
Supplement: Supplementary file 2 — Additional file 2. Questionnaires at 6, 12 and 24 months after vaccination. [file 12882_2022_2680_MOESM2_ESM.pdf]

Questionnaire LESS CoV-2 study

Long term efficacy and safety of SARS-CoV-2 vaccination in kidney patients

Participation number	
Date	

Would you please fill in and return this questionnaire in the return envelope within 7 days after receiving the questionnaire? (stamp is not required).

Questions?

You can find answers to frequently asked questions on the page of www.recovac.nl. If you cannot find your answer, please send an e-mail to info@recovac.nl or call +3150-3610881 (available Monday to Thursday from 9AM to 12AM).

Instructions for answering the questions

With this questionnaire we would like to know if you have been through COVID-19, which corona vaccine has been given to you and if you have experienced any side effects after vaccination. Besides, we would like to ask you questions regarding your health. For example, we would like to know whether you use any kind of medication and if you have been diagnosed with diseases other than kidney disease. You can answer most questions by choosing from several options. Please fill in the option that applies to your situation.

- Answer all questions. You can skip a question only if it is specifically stated that the question does not apply to you.
- Per question you can fill in one option. You can choose more options only when it is specifically stated that you are allowed to do so.
- If an answer option is followed by dots, you are expected to fill in your answer there.
- Please use a pen with black or dark blue ink.
- To be clear, this is not a test; there are no ‘correct or ‘wrong’ answers.

The following questions are about the period after your vaccination and after completing the first questionnaire (1 month after vaccination)

1. Have you been tested positive for the coronavirus?
   - ☐ Yes
   - ☐ No — *Please proceed to question 8*

2. When have you been tested positive for the coronavirus? (mm/yyyy)?

   ____ / ____

3. Where were you tested?
   Multiple answers can apply
   - ☐ Municipal Health Services
   - ☐ Hospital
   - ☐ Commercial provider
   - ☐ General practitioner
   - ☐ At home – self test
   - ☐ Other, …..
   - ☐ Do not know

4. With what kind of test have you been tested?
   - ☐ An extensive test (PCR-test), of which I have received a result within 1-2 days.
   - ☐ A self-test (antigen test), of which I have received a result within minutes to hours.
   - ☐ Do not know.

5. At the time of coronavirus infection, did you have any symptoms?
   - ☐ Yes
   - ☐ No

6. Have you been admitted to the hospital because of a coronavirus infection.
   - ☐ Yes
   - ☐ No
   - ☐ I have been admitted because of another reason and have been tested positive for the coronavirus during admission.

7. Have you been admitted to the ICU because of a coronavirus infection?
   - ☐ Yes
   - ☐ No
   - ☐ I have been admitted because of another reason and have been tested positive for the coronavirus during admission.

8. Have you been admitted to the hospital because of reason other than a coronavirus infection?
   - ☐ Yes
   - ☐ No — *Please proceed to question 20*

9. If yes, have you been admitted to the hospital more than once?
    - ☐ Yes
    - ☐ No

10. If yes, when have you been admitted? (admission 1) (mm/yyyy) ____ / ____

11. For what reason have you been admitted? (admission 1)
    Multiple answers can apply
    - ☐ An infection (other than the coronavirus)
    - ☐ Heart and vascular disease (for example heart attack or stroke)
    - ☐ Surgery / planned operation
    - ☐ Other, …..

12. If yes, when have you been admitted? (admission 2) (mm/yyyy) ____ / ____

13. For what reason have you been admitted? (admission 2)
    Multiple answers can apply
    - ☐ An infection (other than the coronavirus)
    - ☐ Heart and vascular disease (for example heart attack or stroke)
    - ☐ Surgery / planned operation
    - ☐ Other, …..

14. If yes, when have you been admitted? (admission 3) (mm/yyyy) ____ / ____

15. For what reason have you been admitted? (admission 3)
    Multiple answers can apply
    - ☐ An infection (other than the coronavirus)
    - ☐ Heart and vascular disease (for example heart attack or stroke)
    - ☐ Surgery / planned operation
    - ☐ Other, …..

16. If yes, when have you been admitted? (admission 4) (mm/yyyy) ____ / ____

17. For what reason have you been admitted? (admission 4)
    Multiple answers can apply
    - ☐ An infection (other than the coronavirus)
    - ☐ Heart and vascular disease (for example heart attack or stroke)
    - ☐ Surgery / planned operation
    - ☐ Other, …..

18. If yes, when have you been admitted? (admission 5) (mm/yyyy) ____ / ____

19. For what reason have you been admitted? (admission 5)
    Multiple answers can apply
    - ☐ An infection (other than the coronavirus)
    - ☐ Heart and vascular disease (for example heart attack or stroke)

- ☐ Surgery / planned operation
- ☐ Other, …..

20. After you have been vaccinated, have you been diagnosed with one of the following diseases?
    - ☐ Coronary infarction or previous stent placement or bypass surgery of the heart
    - ☐ Blood vessel disease of the abdomen artery (aorta) and/or legs
    - ☐ Heart failure
    - ☐ Diabetes
    - ☐ Hypertension
    - ☐ Cancer, treated with chemo- or immunotherapy in the past five years
    - ☐ Stroke
    - ☐ Dementia
    - ☐ Lung diseases, such as emphysema, asthma or COPD
    - ☐ Liver cirrhosis
    - ☐ HIV/aids
    - ☐ No, none of the above.

21. Do you use any of the following medicine?
    Multiple answers can apply
    - ☐ Antihypertensive medicine
    - ☐ Blood sugar lowering medicine
    - ☐ Prednisolone
    - ☐ Mycophenalate Mofetil (also referred to as CellCept, Myfortic, Myfenax)
    - ☐ Azathioprin (also referred to as Imuran)
    - ☐ One of the following medicines: tacrolimus (also referred to as Adport, Advagraf, Dailiport, Envarsus, Modigraf, Prograft) or ciclosporin (also referred to as Neoral)
    - ☐ One of the following medicines: everolimus (Certican) or sirolimus (Rapamune)
    - ☐ No, none of the above

<u>The following part is about your kidney disease</u>

22. Which situation applies to you since you last filled in the questionnaire.
    - ☐ I have a kidney transplantation and I do not dialyse anymore.
      Continue to question 38
    - ☐ I have had a kidney transplantation and I am currently a dialysis patient.
      Continue to question 43
    - ☐ I am currently a dialysis patient and I have never had a kidney transplantation.
      Continue to question 43
    - ☐ I have a kidney function less than 30% and I have never had a kidney transplantation.
      Continue to question 46.

<u>The following questions apply if you have a kidney transplantation and if you did not receive dialysis treatment</u>

23. Since the last questionnaire you have filled in, have you been transplanted again?
    - ☐ Yes
    - ☐ No *Please proceed to question 25*

24. If yes, when? (dd/mm/yyy) ___ / ___ / ___
    If you cannot recall the exact date, please fill in only the month and the year of kidney transplantation.

25. Since the last questionnaire you have filled in, have you started dialysis treatment?
    - ☐ Yes, haemodialysis
    - ☐ Yes, peritoneal dialysis
    - ☐ No *Please proceed to question 27*

26. If yes, when did you start dialysis treatment? (dd/mm/yyyy) ___ / ___ / ___
    If you cannot recall the exact date, please fill in only the month and the year in which you started dialysis treatment.

27. After filling in the last questionnaire, did rejection of your kidney transplant occur?
    - ☐ Yes
    - ☐ No

Please proceed to question 35

<u>The following questions apply to you if you were a dialysis patient, since last filling in the questionnaire</u>

28. Since last filling in the questionnaire, did anything change in your dialysis modality?
    - ☐ Yes, I have been treated with haemodialysis and recently I have switched to peritoneal dialysis.
    - ☐ Yes, I have been treated with peritoneal dialysis and recently I have switched to haemodialysis.
    - ☐ No

29. Since last filling in the questionnaire, did you receive a kidney transplantation.
    - ☐ Yes
    - ☐ No *Please proceed to question 35*

30. If yes, when? (dd/mm/yyyy) ___ / ___ / ___
    If you cannot recall the exact date, please fill in only the month and the year of kidney transplantation.

    Please proceed to question 35

<u>The following questions apply to you if you were not being treated with dialysis and if you didn't have a kidney transplant, since last filling in the questionnaire</u>

31. Since last filling in the questionnaire, have you received a kidney transplantation?
    - ☐ Yes
    - ☐ No *Please proceed to question 33*

32. If yes, when? (dd/mm/yyyy) ___ / ___ / ___
    If you cannot recall the exact date, please fill in only the month and the year of kidney transplantation.

33. Since the last questionnaire you have filled in, have you started dialysis treatment?
    - ☐ Yes, I have been treated with haemodialysis and recently I have switched to peritoneal dialysis.
    - ☐ Yes, I have been treated with peritoneal dialysis and recently I have switched to haemodialysis.
    - ☐ No

34. If yes, when? (dd/mm/yyyy) ___ / ___ / ___
    If you cannot recall the exact date, please fill in only the month and the year in which you started dialysis treatment.

<u>End of the questionnaire</u>

35. Date of filling in this questionnaire (dd/mm/yyyy) ___ / ___ / ___
